# Upregulation of RBFOX1 in the malformed cortex of patients with intractable epilepsy and in cultured rat neurons

**DOI:** 10.3892/ijmm.2015.2061

**Published:** 2015-01-02

**Authors:** MING WEN, YONG YAN, NING YAN, XIAO SHAN CHEN, SHI YONG LIU, ZHAN HUI FENG

**Affiliations:** 1 Department of Neurology, The First Affiliated Hospital of Chongqing Medical University, Chongqing Key Laboratory of Neurology, Chongqing 400016, P.R. China; 2 Department of Neurology, University-Town Hospital of Chongqing Medical University, Chongqing 401331, P.R. China; 3 Department of Neurology, Xi’an Central Hospital, Xi’an 710003, P.R. China; 4 Department of Neurosurgery, Xinqiao Hospital, Third Military Medical University, Chongqing 400037, P.R. China; 5 Department of Neurology, Affiliated Hospital of Guiyang Medical University, Guiyang 550004, P.R. China

**Keywords:** RNA-binding Fox 1, epilepsy, malformed cortex, neuronal hyperexcitation

## Abstract

Mutations in RNA-binding Fox 1 *(RBFOX1)* are known to be associated with neurodevelopmental disorders including epilepsy, mental retardation and autism spectrum disorder. The deletion of the *Rbfox1* gene in mice has been shown to result in heightened susceptibility to seizures. However, other studies have revealed mutations or the downregulation of *RBFOX1* in specimens obtained from patients with epilepsy or malformations of cortical development (MCD). Generally, the expression of *RBFOX1* varies according to tissue type. In this study, we demonstrated the upregulation of RBFOX1 protein in the cortex of patients with MCD and intractable epilepsy. Electrophysiological recordings of cultured rat cortical neurons with increased Rbfox1 expression also revealed a significantly increased amplitude of action potential (AP) and Na^+^ current density. Some of these neurons (26.32%) even displayed spontaneous, recurrent, epileptiform discharges (SREDs). Additionally, certain Rbfox1 target transcripts associated with epilepsy, including glutamate receptor, ionotropic, N-methyl D-aspartate 1 [*Grin1*, also known as N-methyl-D-aspartate receptor subunit NR1 (NMDAR1)], synaptosomal-associated protein, 25 kDa (SNAP-25 or *Snap25*) and sodium channel, voltage gated, type VIII, alpha subunit (*Scn8a,* also known as Nav1.6) were identified to be upregulated in these cultured cortical neurons with an upregulated Rbfox1 expression. These data suggest that the upregulation of RBFOX1 contributes to neuronal hyperexcitation and seizures. The upregulation of NMDAR1 (*Grin1*), SNAP-25 (*Snap25*) and *Scn8a* may thus be involved in Rbfox1-related neuronal hyperexcitation.

## Introduction

Epilepsy is one of the most common neurological disorders and nearly a quarter of patients with seizures have intractable epilepsy, which is also referred to as ‘medically refractory/drug-resistant’ or ‘pharmacoresistant’ (1). Malformations of cortical development (MCD) are often observed in patients with intractable epilepsy (2). In our previous observations, we collected 3 surgical specimens from patients with intractable epilepsy and MCD. Using Illumina BeadChip technology, we identified >400 abnormally expressed genes in the epileptogenic zone (unpublished data). The RNA-binding Fox 1 *(Rbfox1)* gene is one of the intriguing abnormally expressed genes.

Mutations in *RBFOX1* [also known as ataxin 2-binding protein 1 (A2BP1)] have been observed in a growing number of neurodevelopmental disorders, including epilepsy, mental retardation (3,4) and autism spectrum disorder (5,6). RBFOX1 can regulate both splicing and transcriptional networks in human neuronal development, and can control neuronal excitation (7-9). Alternative splicing is the process of removing introns from pre-mRNA transcripts and joining exons in different combinations (10). There is increasing evidence indicating that alternative splicing affects neuronal development, controls functions in the mature brain and plays important roles in a number of neurological disorders (11). Rbfox1 regulates alternative splicing of neuronal transcripts by binding the sequence (U) GCAUG in introns flanking alternative exons (12,13). Target transcripts of Rbfox1, include those encoding N-methyl D-aspartate 1 [*Grin1*, also known as N-methyl-D-aspartate receptor subunit NR1 (NMDAR1)], synaptosomal-associated protein, 25 kDa (SNAP-25 or *Snap25*) and sodium channel, voltage gated, type VIII, alpha subunit (*Scn8a,* also known as Nav1.6) have been implicated in epileptogenesis (9).

In 2011, Gehman *et al* found that the stimulus intensities required to evoke field excitatory post-synaptic potentials (fEPSPs) in the Rbfox1*^−/−^* brain were lower than those required for the wild-type brain; thus, the authors came to the conclusion that the deletion of *Rbfox1* in mice was responsible for neuronal hyperexcitation (9). Clinical studies have revealed mutations (3–5) or the downregulation (5) of *RBFOX1* in the specimens obtained from patients with epilepsy or MCD; specifically, the specimens were not from the actual epileptogenic lesions. However, the expression of *RBFOX1* varies according to tissue type (12,14). Thus, it is necessary to re-examine the expression of RBFOX1 in cortical lesions from patients with MCD and epilepsy.

It has been suggested that the RBFOX1 protein plays a major role in the cellular response to hyperexcitation. To test this hypothesis, we altered the expression of Rbfox1 in cultured cortical neurons and measured their electrophysiological properties using whole-cell patch clamp recordings. Some well known Rbfox1 target transcripts which have been directly linked to epilepsy were also investigated.

## Materials and methods

### Subjects

We recruited 15 patients with MCD and intractable epilepsy and documented their brain malformations using MRI. All patients underwent pre-operative clinical evaluations, including seizure charts, MRIs, a 24-h electroencephalogram or a video electroencephalogram, sphenoidal electrode monitoring and intraoperative electrocorticography. An intractable seizure was defined as the ‘failure of adequate trials of 2 tolerated and appropriately selected and used anti-epileptic drug schedules to achieve sustained seizure freedom’. Surgical specimens were obtained from these patients at the First Affiliated Hospital of Chongqing Medical University and Xinqiao Hospital of the Third Military Medical University (both in Chongqing, China). We detected the expression of doublecortin (DCX) and tuberous sclerosis (TSC)1/TSC2 in all the specimens by immunohistochemistry. Samples without an abnormal expression of DCX and TSC1/TSC2 were included in this study. A summary of the clinical characteristics of all the patients is shown in Table I.

The control specimens were obtained from 7 patients who underwent neurosurgical intervention due to brain trauma at the Neurosurgical Department of the First Affiliated Hospital of Chongqing Medical University and Xinqiao Hospital of the Third Military Medical University. The surgical specimens were not from the contusion area. The normal brain tissues were resected as these patients had sustained severe craniocerebral injury (SCCI) and internal decompression treatments were unavoidable. None of the specimens were exposed prior to the neurosurgical intervention. All the specimens were resected within 1 h from injury. None of the patients had ever suffered from epilepsy until the neurosurgical intervention. There were no abnormalities observed in the specimens from the control patients. The clinical characteristics of the controls are presented in Table II.

The resected tissues from the parietal lobe were accepted for study. All the specimens were washed with cold 0.9% saline to erase superficial blood. Subsequently, one section from each specimen was fixed in 4% paraformaldehyde immediately after being resected, and the other section was preserved in liquid nitrogen.

### Ethics statement

The present study was approved by the Ethics Committee on Human Research at Chongqing Medical University and written informed consent was obtained from the patients or their relatives in accordance with the Helsinki Declaration.

### Cortical neuronal cultures and lentiviral transfection

All animal procedures were approved by the Commission of Chongqing Medical University for the ethics of experiments on animals and were carried out in accordance with the National Institutes of Health Guide for the Care and Use of Laboratory Animals (NIH publications nos. 80*–*23) revised in 1996. The neocortex was dissected from Sprague-Dawley rat pups on postnatal age (P)0 as previously described (15,16). The neocortex was dissected from Sprague-Dawley rat pups on P0, transferred to D-Hank’s solution (HyClone, Logan, UT, USA) and cut into small sections (~1 mm^3^) using eye scissors. The tissue sections were trypsinized in 4 ml solution (0.125%, w/v) for 10 min at 37°C, and filtered through a mesh screen (39 *μ*m) to obtain a single-cell suspension, followed by centrifugation at 1,000 × g for 5 min at 25°C. The isolated neurons were then seeded in poly-L-Lysine-coated 24-well plates at 5*–*8×10^5^ cells/well. The neurons were cultured in DMEM/F12 medium supplemented with 10% fetal bovine serum and 1% penicillin-streptomycin (all from Gibco, Carlsbad, CA, USA). After 6*–*8 h of incubation, the medium was changed to DMEM/F12 supplemented with 2% B27 (Gibco) and 1% penicillin-streptomycin. The cells were cultured in an incubator at 37°C with circulating air and 5% carbon dioxide and the medium was changed every 3*–*4 days. All efforts were made to minimize the number of animals used and any possible suffering caused to them.

Lentiviral-mediated-α-Rbfox1 (Rbfox1) and the control lentivirus (lentivirus) were synthesized at Shanghai GeneChem Co., Ltd. (Shanghai, China; nos. V20130406004 and V20130303005). Following 72 h of incubation, the lentiviral-mediated-α-Rbfox1 and the control lentivirus media were added to the cultured neurons with a multiplicity of infection (MOI) of 5. Untransfected cells were used as controls. To calclulate the amount of virus required for a certain MOI, the following formula was used: total ml of virus required = cells × desired MOI/(plaque forming units/ml). All the media was changed 12 h later. Four days after transfection, RT-qPCR, western blot analysis, immunofluorescence assay and whole cell patch-clamp recordings were performed.

### Immunohistochemistry

Immunohistochemical analyses were performed using a previously published streptavidin-biotin-peroxidase complex method (17). Paraffin sections were dried at 60°C for 20 min, dewaxed in xylene, rehydrated through a graded series of alcohol and immersed in 3% hydrogen peroxide for 15 min to block endogenous peroxidase activity. The sections were then blocked with normal non-immune goat serum and incubated with anti-Rbfox1 antibody (no. ab94581, 1:200; Abcam, Cambridge, UK) at 4°C overnight. Subsequently, the sections were incubated with biotin-labeled secondary antibody at a 1:500 dilution (no. A0277; Beyotime Institute of Biotechnology, Shanghai, China) for 1 h at room temperature and stained with 3,3-diaminobenzidine (DAB; Zhongshan Golden Bridge Biotechnology Co., Ltd., Beijing, China) after washing with phosphate-buffered saline (PBS) again. Finally, the sections were counterstained with haematoxylin, dehydrated and mounted. PBS was used to replace the primary antibody as a negative control. Three types of MCD, including focal cortical dysplasia (FCD), TSC and double cortex (DC) were examined. The sections were observed and images were acquired using an Olympus BX51 microscope fitted with a digital camera and DP Controller software (Olympus, Tokyo, Japan).

### Immunofluorescence

Immunofluorescence assay was performed to examine the expression of Rbfox1 and its target transcripts. The cells were washed twice with ice-cold PBS and fixed in 4% paraformaldehyde for 15 min at room temperature. After washing with PBS 3 times, the cells were incubated with 0.2% Triton X-100 for 15 min. To block nonspecific binding, the cells incubated with 5% albumin from bovine serum (BSA) at room temperature for 30 min, followed by incubation with a primary antibody at 4°C overnight. The cells were then incubated with a secondary antibody for 30 min at 37°C in a humidified atmosphere. After washing 3 times with PBS, the nuclei were stained with DAPI (no. C1005; Beyotime Institute of Biotechnology). The following primary antibodies were used: α-Rbfox1 (no. ab94581, 1:1,000; Abcam), microtubule-associated protein 2 (MAP2; no. sc-74421, 1:100; Santa Cruz Biotechnology Inc., Delaware, CA, USA), Nav1.6 (no. ab65166, 1:1,000), NMDAR1 (no. ab28669, 1:1,000) and SNAP25 (no. ab24732, 1:1,000) (all from Abcam). DyLight 649-conjugated goat anti-rabbit IgG (1:100) and DyLight 649-conjugated goat anti-mouse IgG (1:100) (both from CWbiotech, Beijing, China), DyLight 649-conjugated donkey anti-goat IgG (1:100; Jackson ImmunoResearch Laboratories, Inc., West Grove, PA, USA), FITC-conjugated goat anti-rabbit IgG (1:100) and FITC-conjugated rabbit anti-mouse IgG (1:100) (both from Beijing DingGuo ChangSheng Biotechnology Co., Ltd., Beijing, China) were used as the secondary antibodies. Neurons were observed under a laser scanning confocal microscope (TCS-SP2; Leica Microsystems GmbH, Wetzlar, Germany).

### Whole-cell patch clamp recordings

Whole-cell patch clamp recordings were acquired from cortical neurons isolated from the rat neocortex following a 4-day culture using previously established protocols (18). Briefly, a neuronal culture plate was mounted on the stage of an inverted microscope (IX-51; Olympus) and continuously perfused with standard extracellular solution containing 140 mM NaCl, 5 mM KCl, 1 mM MgCl_2_, 2 mM CaCl_2_, 10 mM HEPES, 10 mM D-glucose and 10 mM tetraethyl-ammonium (TEA) Cl (pH 7.4 with CsOH). The experiments were performed at room temperature (22*–*24°C). Recordings were made using patch electrodes with a resistance of 5-10 MΩ. The internal solution contained 130 mM KAsp, 5 mM ATP-Na2, 2 mM MgCl_2_, 1 mM CaCl_2_ and 10 mM HEPES (pH 7.2 with CsOH). The osmolarity was adjusted to 290±10 mOsm with sucrose. The intracellular recordings were carried out using an EPC-10 amplifier (HEKA Elektronik Dr. Schulze GmbH, Lambrecht/Pfalz, Germany) in current clamp mode. The pipette resistance and capacitance were compensated electronically after the establishment of a gigaseal. Following whole-cell capacitance compensation, recordings were made only when the series resistance was <20 MΩ. Cultured neurons with small dendritic arborizations, long axon and soma diameters of 20-26 *μ*m were selected for the electrophysiological recordings to avoid space clamp artifacts. Routinely, 60-80% series resistance compensation was employed, continually monitored and adjusted as required. Whole-cell resistance and resting membrane potential were also monitored before and during the experiments and a cell was accepted for study only if these parameters remained stable.

Spontaneous firing, voltage-gated Na^+^ currents, and evoked action potentials (APs) were measured. Under current-clamp mode, the spontaneous firing of the cultured cortical neurons was firstly recorded. In order to further investigate whether Rbfox1 can influence the excitability of the cultured cortical neurons, the cells were held at −80 mV, and a series of 20 msec depolarizing current pulses in 10 mV increments from −80 to 70 mV was delivered to elicit the cell generating voltage-gated Na^+^ currents. The cells were then held at −70 mV, and a series of 40 msec depolarizing current pulses in 10 pA increments from 0 to 80 pA was delivered to elicit the cell generating evoked APs. Voltage-gated Na^+^ currents were measured from threshold to the Na^+^ currents peak. The AP amplitudes were measured from the threshold to the AP peak. Data were digitized and transferred to videotape using a Clamp-fit 10.0 device (from Axon Instruments, Sunnyvale, CA, USA). Fitting and statistical analysis were performed using Igor4.03 (WaveMetrics, Portland, OR, USA), SPSS 19 (SPSS Inc., Chicago, IL, USA) and Origin7.5 sofware (OriginLab Corp., Northampton, MA, USA).

### Western blot analysis

**Figure 1:**
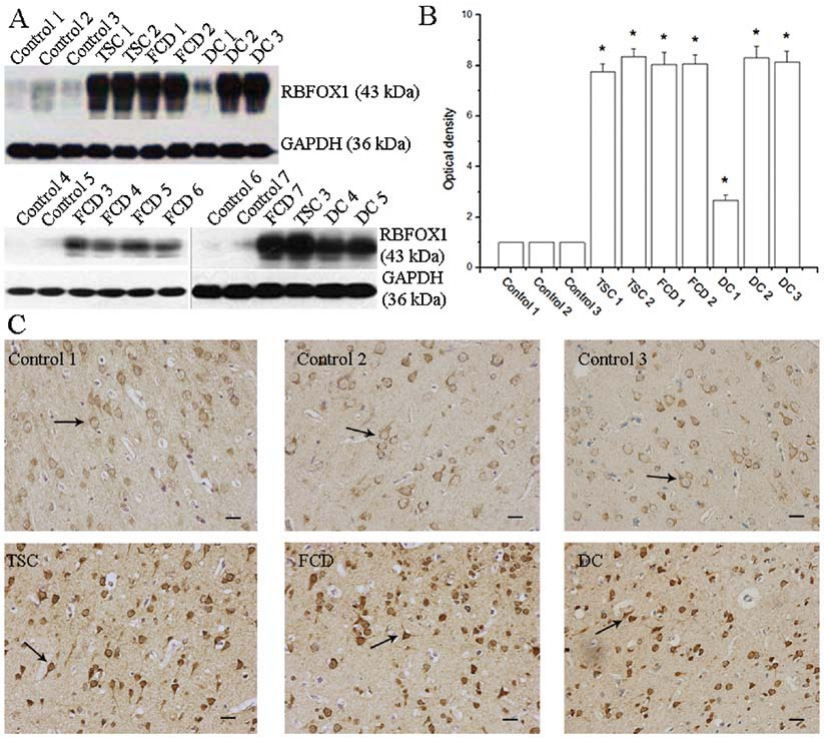
Expression of RNA-binding Fox 1 (RBFOX1) protein in patients with epilepsy/malformations of cortical development (MCD) and controls. (A) Expression of RBFOX1 protein in patients and controls determine by western blot analysis. GAPDH was used as a loading control. (B) Quantification of the representative western blot analysis results. Data represent the means ± SD. RBFOX1 protein expression was significantly upregulated in the epilepsy/MCD group (lanes 4–10) compared to the average level of the controls (lanes 1–3). ^*^P<0.05 was considered to indicate a statistically significant difference. (C) Protein expression of RBFOX1 in patients and controls analyzed by immunohistochemical staining. Top panel, control specimens; bottom panel, specimens from patients with tuberous sclerosis (TSC), focal cortical dysplasia (FCD) and double cortex (DC). Magnification, ×400. Scale bar, 50 *μ*m.

Protein levels in the human cortical tissue and cultured cortical neurons were measured by western blot analysis. We analyzed all the 15 specimens obtained from patients with both MCD and intractable epilepsy and then analyzed the optical density of 7 representative examples, including 2 patients with tuberous sclerosis (lanes/bars 4 and 5), 2 with focal cortical dysplasia (lanes/bars 6 and 7) and 3 with double cortex (lanes/bars 8, 9 and 10) (Fig. 1).

Total protein which was derived from the human cortical tissue and cultured rat cortical neurons was harvested using RIPA lysis buffer containing a protease and phosphatase inhibitor cocktail (KeyGen Biotech Co., Ltd., Nanjing, China). The protein concentrations were measured using the Bradford method (Beyotime Institute of Biotechnology). The protein samples (50 *μ*g) were fractionated by 10% SDS-polyacrylamide gel electrophoresis, electroblotted onto a polyvinylidene difluoride (PVDF; Millipore, Billerica, MA, USA) membrane and immunoblotted with a primary antibody and GAPDH (1:1,000; Beijing DingGuo ChangSheng Biotechnology Co., Ltd.) as an internal control. The following primary antibodies were used: α-Rbfox1 (no. ab94581, 1:1,000; Abcam), Nav1.6 (no. ASC-009, 1:1,000; Alomone, Jerusalem, Israel), NMDAR1 (no. ab28669, 1:1,000) and SNAP25 (no. b24732, 1:1,000) (both from Abcam). Horseradish peroxidase-conjugated secondary antibodies (nos. A0208, A0216 and A0181, 1:1,000; Beyotime Institute of Biotechnology) were used. RBFOX1, Nav1.6, NMDAR1 and SNAP25 proteins were identified as immunopositive bands with molecular weights of 43, 220, 120 and 29 kDa, respectively. The bands were detected using the Pierce ECL western blotting substrate (Thermo Fisher Scientific, Inc., Rockford, IL, USA), scanned with a Bio-rad GS-800™ calibrated densitometer and analyzed using Quantity One software version 4.6.2 (Bio-Rad Laboratories, Hercules, CA, USA).

### RT-qPCR

RT-qPCR was used for *Rbfox1* only. The cultured rat cortical neurons were homogenized prior to RNA extraction in 1 ml of TRIzol. All RNA was quantified using a spectrophotometer (NanoDrop1000; Thermo Fisher Scientific, Inc.) to determine the optical density at 260/280 nm ratios. Reverse transcription was performed on 2 *μ*g RNA using the K1622 RevertAid First Strand cDNA Synthesis kit (Fermentas Canada Co., Ltd., Burlington, ON, Canada) according to the manufacturer’s instructions. The primers for rat *α-Rbfox1* (forward, 5′-CTACAGTGACAGTTACGGACGAG-3′ and reverse, 5′-ATGAAGAAAGAACGAGACCC-3′) were purchased from BGI Tech (Shenzhen, China). RT-qPCR was performed using 2X SYBR Mix SsoAdvanced SYBR-Green Supermix (Bio-Rad Laboratories). Amplification was conducted with an initial denature action step at 95°C for 5 min, followed by 95°C for 5 sec, 60°C for 30 sec and 72°C for 15 sec, 40 cycles, 72°C for 10 min and a final melting curve. Gene analysis was carried out in triplicate. *Gapdh* was used as a loading control. The data were collected and analyzed using OneStep Software (Applied Biosystems, Foster City, CA, USA). Relative quantification was performed using the 2^−ΔΔCt^ method, as previously described (19).

### Statistical analysis

All data are expressed as the means ± SD. An independent samples t-test was used for 2 sample comparison. One-way analysis of variance (ANOVA) followed by Dunnett t (2 sided) test or Dunnett’s T3 test was used for multiple comparisons of treated/patient groups against the controls. Linear fits and statistical analyses were performed using Igor 4.03 (WaveMetrics), SPSS 19 (SPSS Inc.) and OriginPro 7.5 sofrware (OriginLab Corp.) software. A value of P<0.05 was considered to indicate a statistically significant difference.

## Results

### Patient profiles

A total of 15 individuals (8 females and 7 males) aged from 11 to 36 years with both MCD and intractable epilepsy were recruited in this study. In total, 7 patients had focal cortical dysplasia, 5 had a double cortex and 3 had tuberous sclerosis. No altered expression of the *DCX* or *TSC1/TSC2* genes was observed in the 15 specimens obtained from these patients.

Control samples were obtained from 7 brain trauma patients who underwent neurosurgical intervention. There were no histopathological abnormalities observed in these patients (Table II). There was no statistically significant difference in the mean age between the controls and the patients with MCD/epilepsy [t_0.05/2(14,4)_=0.363, P=0.721, independent samples t-test].

### Protein expression of RBFOX1 in patients

The expression of RBFOX1 protein in tissues from both the controls and patients with MCD and intractable epilepsy was analyzed by western blot analysis and immunohistochemistry assays. The results from western blot analysis revealed that the protein expression of RBFOX1 was markedly upregulated, not only in the patients with double cortex, but also in patients with focal cortical dysplasia and tuberous sclerosis (Fig. 1A). There was a significant difference in the RBFOX1 protein level between the patients with epilepsy/MCD and the controls [F_(9,20)_=199.855, P=0.000, ANOVA]. ANOVA followed by the Dunnett t (2 sided) test was used for multiple comparisons of the protein levels in the patients with epilepsy/MCD compared to the average levels of the controls (lanes 1*–*3). The results revealed a significant difference in the protein levels in the epilepsy/MCD group (P=0.004, 0.004, 0.009, 0.005, 0.041, 0.008 and 0.007 for bars 4–10, respectively) against the average of the controls (bars 1–3; Fig. 1B). Specimens from patients with double cortex, focal cortical dysplasia and tuberous sclerosis were analyzed by immunohistochemistry. A significantly stronger RBFOX1 staining signal was observed in the cortical lesions of the patients with MCD/epilepsy compared to the controls (Fig. 1C). In summary, these data demonstrate the overexpression of RBFOX1 protein in the cortical lesions of patients with MCD and intractable epilepsy.

### Assessment of transfection efficiency

**Figure 2:**
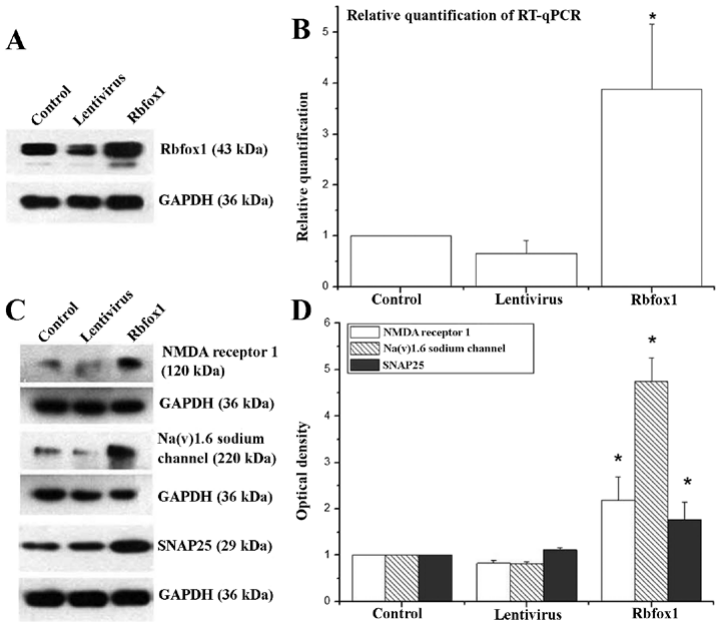
Expression of Rbfox1, NMDA receptor 1, Na(v)1.6 sodium channel and SNAP25 in cultured neurons. GAPDH was used as a loading control. Data represent the means ± SD. ^*^P<0.05 was considered to indicate a statistically significant difference. (A) Expression of Rbfox1 in cultured neurons analyzed by western blotting. (B) Rbfox1 RNA level analyzed by RT-qPCR and quantification of the western blotting results. ^*^Expression of Rbfox1 was markedly increased in the Rbfox1 group compared to the controls. (C) Expression of NMDA receptor 1, Na(v)1.6 sodium channel and SNAP25 in cultured neurons determined by western blot analysis. (D) Quantification of the western blot analysis results. Expression of NMDA receptor 1, Na(v)1.6 sodium channel and SNAP25 was significantly increased in Rbfox1 group compared to the control group. ^*^P<0.05 was considered to indicate a statistically significant difference. The control group consisted of untransfected cells.

The transfection efficiency was assessed 4 days following transfection by western blot analysis, RT-qPCR and immunofluorescence staining. Western blot analysis (Fig. 2A) and immunofluorescence staining (Fig. 4) revealed a significant upregulation of Rbfox1 expression in the lentiviral-mediated-Rbfox1 transfected neuron group (Rbfox1 group). As shown by western blot analysis, there was a significant difference in the Rbfox1 protein level between the Rbfox1 group, the control-lenti-viral-vector transfected neurons (lentivirus group) and the untransfected group (control group) [F_(2,6)_=220.821, P=0.000, ANOVA]. ANOVA followed by Dunnett’s T3 test was used for multiple comparisons of the Rbfox1 protein level in the 3 groups. Statistical analysis revealed a significant difference between the Rbfox1 group (P=0.010) and the control group; however, no significant difference was observed between the lentivirus group (P=0.056) and the control group. RT-qPCR analysis (Fig. 2B) revealed that the *Rbfox1* mRNA level was 387 and 63% of the controls in the Rbfox1 group and lentivirus group, respectively. There was a significant difference in the *Rbfox1* mRNA level between the Rbfox1 and lentivirus and the control groups [F_(2,6)_=16.242, P=0.004, ANOVA]. ANOVA followed by the Dunnett t (*2* sided) test was used for multiple comparisons of the mRNA levels in the 3 groups. The results revealed a significant difference in *Rbfox1* mRNA levels between the Rbfox1 group (P=0.006) and the control group; however, no significant difference was observed between the lentivirus group (P=0.812) and the control group.

### Whole-cell patch clamp recordings

**Figure 3:**
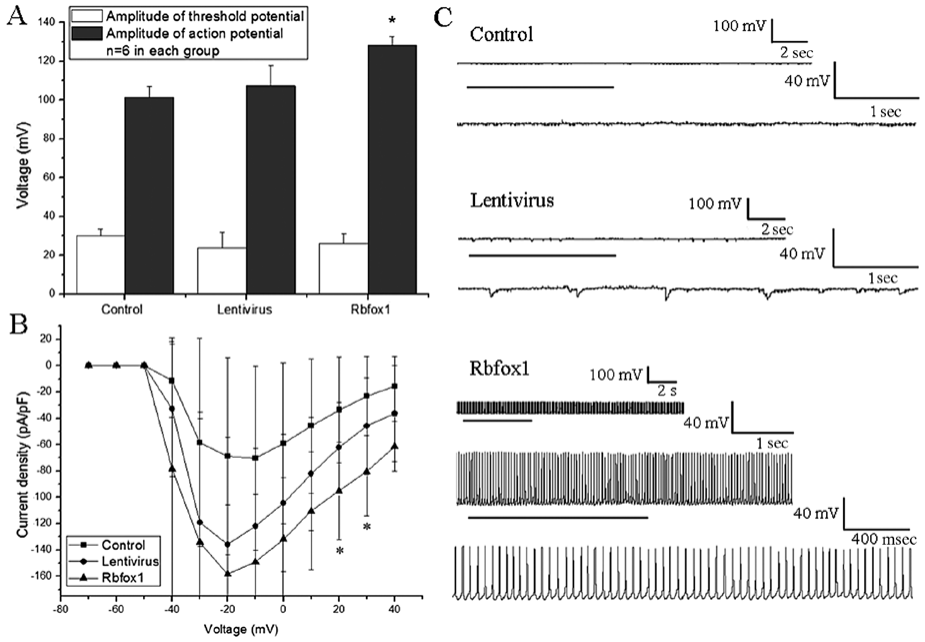
Whole-cell patch clamp recordings. The lentivirus group was used as a reference. Data represent the means ± SD. ^*^P<0.05 was considered to indicate a statistically significant difference. (A) The amplitude of threshold potential and action potential of cultured neurons. The amplitude of action potentials in the Rbfox1 group was markedly increased compared to the lentivirus group. No statistically significant difference in threshold potential was observed between the lentivirus and the control groups. (B) The Na^+^ current density-voltage curves of cultured neurons. Statistical analysis revealed a significant difference between the Rbfox1 group and the lentivirus group at 20 and 30 mV. (C) Spontaneous discharge of a lentiviral-mediated Rbfox1-transfected neuron. Enlarged trace showing evoked epileptiform event manifested high-frequency, spontaneous, recurrent, epileptiform discharges (SREDs). No SREDs were observed in the control and lentivirus groups.

We measured the absolute value of threshold potential, AP and sodium currents in each group (n=6). Spontaneous discharges were also recorded in each group. The lentivirus group was used as a reference. There was no statistically significant difference observed in membrane capacitance [F_(2,15)_=2.889, P=0.061, ANOVA] or threshold potential between the Rbfox1, lentivirus and control groups [F_(2,15)_=2.022, P=0.167, ANOVA]. Statistical analysis revealed a significant difference in AP between the 3 groups [F_(2,15)_=22.115, P=0.000, ANOVA, Fig. 3A]. ANOVA followed by the Dunnett t (2-sided) test was used for multiple comparisons of the AP between the 3 groups. The amplitude of APs in the Rbfox1 group was markedly increased compared to the lentivirus group (P=0.000). No significant difference was observed however, between the untransfected (control) group and the lentivirus group (P=0.307).

No statistical difference was observed in the Na^+^ current density when the step voltage was from −40 to 10 mV [F_(2,15)_=1.578, 1.314, 2.626, 2.631, 2.813, 2.924, respectively, P=0.239, 0.298, 0.105, 0.105, 0.092, 0.085, respectively, ANOVA] and 40 mV [F_(2,15)_=3.399, P=0.061, ANOVA]. Statistical analysis revealed a significant difference in the Na^+^ current density between the 3 groups when the step voltage was 20 or 30 mV [F_(2,15)_=4.093 and 4.441, P=0.038 and 0.031, respectively, ANOVA, Fig. 3B]. ANOVA followed by the Dunnett t (2-sided) test was used for multiple comparisons of the Na^+^ current density between the Rbfox1 and untransfected groups against the lentivirus group. Statistical analysis revealed a significant difference between the Rbfox1 group and the lentivirus group at 20 and 30 mV (P=0.022 and 0.018, respectively). No significant difference was observed between the untransfected group and the lentivirus group at 20 and 30 mV (P=0.246 and 0.165, respectively).

In the case of spontaneous discharges, there were no spontaneous, recurrent, epileptiform discharges (SREDs) observed in the untransfected and lentivirus groups. This epileptiform activity was characterized by abruptly developing, repetitive high-frequency burst spiking that overlaid a paroxysmal depolarization shift (20,21). In the Rbfox1 group, 5 (26.32%) out of 19 neurons displayed SREDs (Fig. 3C).

Overall, these data suggest that changes in the Rbfox1 protein level result in changes in AP amplitude and SREDs, and cause a generalized increase in the excitability of cultured cortical neurons.

### Identification of Rbfox1 target transcripts involved in epilepsy

**Figure 4:**
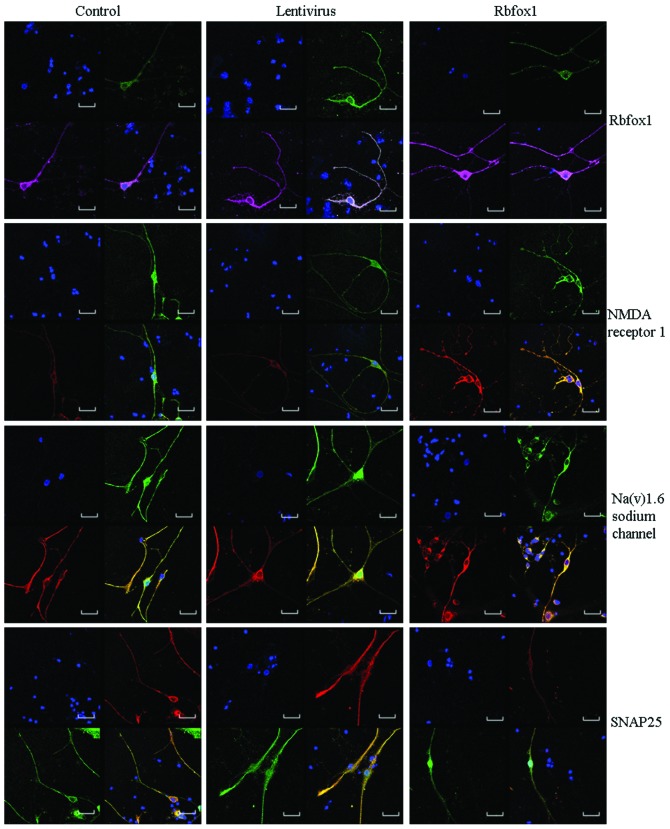
Expression of Rbfox1, NMDA receptor 1, Na(v)1.6 sodium channel and SNAP25 in cultured neurons analyzed by immunofluorescence staining. Double-label fluorescent immunocytochemistry for the simultaneous detection of Rbfox1 (magenta), NMDA receptor 1 (red) and Na(v)1.6 sodium channel (red) with MAP2 (green). Double-label fluorescent immunocytochemistry for the simultaneous detection of SNAP25 (green) with MAP2 (red). All nuclei of the cells and the cell debris were stained with DAPI (blue). Magnification, ×800. Scale bar, 30 *μ*m.

We examined the expression of NMDAR1 (*Grin1*), SNAP-25 (*Snap25*) and *Scn8a* (or Nav1.6) in our transfected cortical neurons by western blot analysis (Fig. 2C) and immunofluorescence staining (Fig. 4). When the Rbfox1 protein level was increased, all 3 proteins were upregulated.

Statistical analysis revealed a significant difference in the levels of the NMDAR1, SNAP-25 and Nav1.6 transcripts between the 3 groups [F(2,6)=19.961, 8.33 and 162.779, respectively, P=0.002, 0.008 and 0.000, respectively, ANOVA, Fig. 2D]. ANOVA followed by the Dunnett (2-sided) test was used for multiple comparisons between the levels of NMDAR1 and SNAP-25 in the Rbfox1 and lentivirus group and the control group. Statistical analysis revealed a significant difference in the levels of these transcripts between the Rbfox1 group and the control group (P=0.004 and 0.010, respectively). No significant difference was observed however, between the lentivirus group and the control group (P=0.698 and 0.909, respectively). ANOVA followed by the Dunnett’s T3 test was used for multiple comparisons between the levels of Nav1.6 in the Rbfox1 and lentivirus group and the control group. Statistical analysis revealed a significant difference in the Nav1.6 expression level between the Rbfox1 group (P=0.013) and the cotnrol group. No significant difference was observed however, between the lentivirus group (P=0.056) and the control group.

## Discussion

Intractable epilepsy and neurodevelopmental delay are the most common clinical manifestations of MCD (22). Modern advanced imaging techniques have improved our ability to detect MCD in patients with intractable epilepsy (23). Neurosurgical intervention is a potential curative treatment for a subset of patients with intractable epilepsy and MCD (22,24). This makes it possible to explore abnormally expressed genes in the epileptogenic zone. Mutations in *RBFOX1* have been observed in epilepsy or mental retardation, although brain tissues were not available in these studies (3,4). In this study, we provided new evidence to support the hypothesis that abnormally expressed RBFOX1 is related to epilepsy/MCD by analyzing the expression of RBFOX1 in the epileptogenic zone. We demonstrated that RBFOX1 protein is upregulated in cortical lesions obtained from patients with epilepsy/MCD. By electrophysiological recordings analysis, we found that cultured rat cortical neurons with an increased Rbfox1 expression were hyperexcitated, which provides direct evidence to support the hypothesis that Rbfox1 may contribute to neuronal hyperexcitation and epilepsy.

We analyzed the expression of RBFOX1 in brain tissues obtained from patients with epilepsy/MCD and control subjects. Although the patients had different types of MCD, they all overexpressed RBFOX1. In fact, there is a notion that the boundaries between disorders of neuronal proliferation, migration, or cortical organisation are fading, as supported by a recent report of mutations in WD repeat domain 62 (WDR62), dynein, cytoplasmic 1, heavy chain 1 (DYNC1H1) and tubulin, gamma 1 (TUBG1) with a broad range of MCD types (25). Previous studies have suggested that mutations in RBFOX1 are related to epilepsy (3,4). We suspected that the overexpression of RBFOX1 in the epileptogenic zone may contribute to epilepsy. While the deletion of the *Rbfox1* gene has been shown to result in neuronal hyperexcitation and seizures in mice (9), it thus surprised us that in the cortical tissue obtained from patients with intractable epilepsy and MCD, the protein expression of RBFOX1 was increased. However, in their study, Gehman *et al* (9) did not use a transgenic mouse model by increasing the Rbfox1 protein level to explore the effects on neuronal hyperexcitation. Thus, the increased protein level of RBFOX1 in the brain tissues of patients with epilepsy/MCD is a significant finding and worthy of further study. A question which remains unanswered however, is whether the increased RBFOX1 protein expression caused the seizures, or whether the seizures themselves resulted in the increased protein levels of RBFOX1. In order to attempt to address this issue, we investigated neuronal excitation in cultured cortical neurons with different levels of RBFOX1 protein.

We used the lentivirus-mediated transfection technique to alter the Rbfox1 protein level in cultured cortical neurons. The AP, Na^+^ current density and SREDs of these neurons were measured by whole-cell patch clamp recordings. To avoid the possible influence of the lentivirus vector on the electrophysiological recordings, we selected the control lentivirus group as a reference. Our results revealed that the AP amplitude and Na^+^ current density increased as the Rbfox1 protein level increased. In fact, some neurons even displayed SREDs. Based on these data, we concluded that the increased Rbfox1 protein expression may be the cause of hyperexcitation in cultured cortical neurons, and the increased RBFOX1 protein epxression is likely the cause of epilepsy in human patients rather than a symptom of the epilepsy.

Once we established this causal relationship between the altered Rbfox1 protein expression and neuronal hyperexcitation, we then wished to determine whether this increase in excitability results from changes in certain known Rbfox1 target transcripts that have been directly linked to epilepsy. As mentioned above, the expression levels of NMDAR1 (or *Grin1*), the Nav1.6 (or *Scn8a*) and SNAP-25 (or *Snap25*) were elevated in the neurons with an increased Rbfox1 protein expression.

NMDAR1 (or *Grin1*), one of the fundamental excitatory neurotransmitter receptors for basic brain development and function (26), was upregulated in the cultured neurons with an increased Rbfox1 protein expression and this was expected. Both in animal models and patients with epilepsy, the increased expression of NMDAR1 has been reported to be associated with epilepsy (27,28). Nav1.6 is the most abundantly expressed sodium channel in the adult central nervous system and is critical for AP generation and propagation (29). Blumenfeld *et al* found that kindling was associated with increased persistent sodium current density in CA3 neurons and an increased mRNA and protein expression of Nav1.6 in these neurons, suggesting that Nav1.6 may participate in a self-reinforced cycle of kindling (30). APs are initiated by Nav1.6 channels in the axon initial segment based on their low threshold and high channel density (29,31). No matter the excitability of other neurons and neurotransmitters, neurons with an increased Nav1.6 protein level tend to display evoked epileptiform events. In this study, we demonstrated that SREDs and an enhanced protein expression of Nav1.6 may contribute to SREDs in the neurons with an altered Rbfox1 protein expression. This may partially explain why these neurons exist in an epileptogenic zone and are prone to the initial ictal period. SNAP25 is a plasma membrane protein which mediates exocytosis by participating in forming the soluble N-ethylmaleimide factor attachment receptor (SNARE) complex (32). It has been suggested that changes in SNAP25 gene expression may also contribute to epilepsy (33).

Unexpectedly, our findings are inconsistent with those found in the study by Gehman *et al* (9) using Rbfox1 knockout mice. They found that the deletion of the *Rbfox1* gene resulted in increased excitability in the dentate gyrus, and the decreased expression of NMDAR1, Nav1.6 and SNAP-25 (9). One possible explanation for this difference is that their experiments were performed *in vitro* and may represent more immediate changes that are not observed *in vivo*. The increased expression of NMDAR1, Nav1.6 and SNAP-25 in cultured cortical neurons with an increased Rbfox1 protein expression may be involved in Rbfox1-related neuronal hyper-excitation, although further studies are required to confirm these findings.

## Figures and Tables

**Table I:** Clinical characteristics of patients with MCD and intractable epilepsy.

Patient no.	Gender	Age at surgery (years)	Seizure duration (year)	AEDs prior to surgery	Resected regions of cases	Classification
1	F	24	18	CBZ, PHT, VPA, PB	TNr	FCD
2	M	33	10	VPA, CBZ, TPM	TNl	FCD
3	M	28	18	CBZ, VPA, TPM, LEV	TNr	FCD
4	M	36	12	VPA, TPM, LEV, PB, CZP	TNr	FCD
5	F	18	10	CBZ, PHT, TPM, PB	TNr	FCD
6	F	30	12	CBZ, TPM, PB	TNr	FCD
7	M	36	13	CBZ, VPA, TPM	TNl	FCD
8	F	18	17	VPA, OCBZ, CBZ	TNl	TSC
9	M	23	20	CBZ, VPA, TPM, PB	TNl	TSC
10	M	11	9	VPA, OCBZ, CBZ, TPM	TNr	TSC
11	M	34	28	CBZ, PHT, PB	TNr	DC
12	F	20	15	TB, CBZ, VPA, TPM	TNl	DC
13	F	18	12	CBZ, PB, TPM	TNr	DC
14	F	15	6	VPA, CBZ, PB	TNl	DC
15	F	25	22	CBZ, VPA, PHT	TNl	DC

MCD, malformations of cortical development; F, female; M, male; AEDs, anti-epileptic drugs, CBZ, carbamazepine; PHT, phenytoin; VPA, valproate; PB, phenobarbital; TPM, topiramate; LEV, levetiracetam; CZP, clonazepam; OCBZ, oxcarbazepine; TN, temporal neocortex; r, right; l, left; FCD, focal cortical dysplasia; TSC, tuberous sclerosis; DC, double cortex.

**Table II:** Clinical characteristics of the controls.

Patient no.	Gender	Age (years)	Etiology diagnosis	Resection tissue	Adjacent tissue pathology
1	M	33	Trauma	TNl	n
2	M	21	Trauma	TNr	n
3	F	31	Trauma	TNr	n
4	F	14	Trauma	TNr	n
5	F	25	Trauma	TNl	n
6	M	13	Trauma	TNl	n
7	M	26	Trauma	TNl	n

M, male; F, female; TN, temporal neocortex; l, left; r, right; n, normal; FN, frontal neocortex; TN, temporal neocortex; r, right; l, left; n, normal.
